# Tonsillar T-cell lymphoma with hepatic and renal metastases in a fattening domestic pig (*Sus scrofa domesticus*)

**DOI:** 10.1186/s12917-025-05229-2

**Published:** 2026-01-07

**Authors:** Giuseppe Giglia, Lucia Minelli, Nicoletta D’Avino, Alice Ranucci, Elvio Lepri, Elisabetta Manuali

**Affiliations:** 1 Department of Veterinary Medicine, University of Perugia, Via S. Costanzo 4, Perugia, 06126 Italy; 2 Istituto Zooprofilattico Sperimentale dell’Umbria e delle Marche “Togo Rosati”, Via G. Salvemini 1, Perugia, 06126 Italy

**Keywords:** *Sus scrofa domesticus*, Soft palate, Tonsil, Epitheliotropic lymphoma, Immunohistochemistry

## Abstract

**Background:**

In pigs, lymphoma is documented only sporadically in literature. Compared with other livestock species, such as cattle, lymphoma in pigs has a significantly lower incidence. The low number of reported cases may be due in part to underdiagnosis related to the production environment in which these animals are raised, together with the sporadic and non–virus-associated nature of the disease. Although rare, lymphomas represent the most frequent form of hematopoietic tumor in this species with different sites of development. To date, there are no clear reports of primary tonsillar lymphoma in pigs in the literature.

**Case presentation:**

This case report describes a rare case of tonsillar lymphoma with liver and kidney metastases in an intensively reared male castrated Danish pig. The animal, apparently in good health, was found dead at the age of 4 months (120 days), without any premonitory clinical signs. On necropsy, enlargement of the tonsils of the soft palate, with firm consistency and red color and multiple white-to-gray nodules in the liver and kidneys were observed, and considered consistent with a round cell tumor. Histopathological and immunohistochemical analyses identified a T cell lymphoma with primary tonsillar localization and metastatic spread.

**Conclusions:**

Compared with other livestock species, such as cattle, lymphoma in pigs has a significantly lower incidence and this report contributes to expanding knowledge about the anatomical distribution and biological behavior of lymphomas in pigs. This case highlights the importance of including histologic examination of the tonsils in postmortem investigations, especially in the presence of metastatic lesions of uncertain origin. It also suggests the usefulness of implementing systematic reporting systems for neoplastic lesions in pigs to improve health surveillance and deepen the epidemiology of neoplasms in the swine species. Increased awareness among farm veterinarians, inspectors, and diagnosticians can help refine early diagnosis and health management on the farm.

## Background

 Lymphoma is a malignant neoplasm originating from lymphoid tissues and is considered the most common hematopoietic tumor in swine [1–5]. Although it remains a relatively rare malignancy in pigs compared to other farm animal species, lymphoma has been sporadically reported in pigs of various breeds, ages, and sex [1–3, 5–14]. In swine, lymphoma has been identified in multiple anatomical locations, such as the mediastinum, thymus, mesenteric lymph nodes, spleen, and even extra-nodal sites such as the kidneys and liver, the two latter often as metastatic events [1, 2, 7, 9, 10, 3, 15, 16]. The pathogenesis of lymphoma in pigs is not yet fully elucidated, but reported cases have suggested a potential hereditary basis [17–19], particularly where familial clusters of lymphomas have been observed in specific pig breeds. Additionally, the lack of reports of cases with an enzootic development tend to exclude a direct viral oncogenesis in pigs. Regarding the age of development, historically, Anderson and colleagues reported 92 cases, classified into multicentric and thymic, also in young individuals (3 to 6 months of age) [2]. Similarly, also Hayashi and colleagues reported 16 cases predominantly in pigs less than one year old, further confirming the statement that lymphomas can also arise in young animals [20]. Migaki and colleagues documented 200 lymphoma’s cases, noting a metastatic pattern with frequent metastases to liver and kidney [6]. Fisher and colleagues noted that many tumors, including multicentric and visceral forms, are present in the absence of clinical signs [7]. Additional cases described later identified forms of plasmacytic differentiation with immunoglobulin cytoplasmic inclusions [3, 11, 13, 14]. One of these cases, a multicentric lymphoma, also involved the tonsil [3]. Immunohistochemistry and molecular analysis are critical for accurate classification and subtype identification, yet these are not routinely performed in farm animal cases due to practical and cost limitations. Nonetheless, the increasing popularity of pet pigs and the use of swine as biomedical models have underscored the need for deeper understanding of this disease in the porcine species. Developing pig models of lymphoma could help compare pathogenesis and treatment modalities across species, given the physiological and immunological similarities between pigs and humans.

The aim of this report is to highlight the occurrence of a primary tonsillar T-cell lymphoma in a young pig with hepatic and renal metastases, thereby increasing awareness among veterinarians, researchers, and pig owners. Improved recognition, early diagnosis, and systematic documentation of such cases are essential not only for animal welfare and pig management, but also for the potential development of porcine models to study human lymphomas.

## Case presentation

The examined animal was a 80 kg, Danish, castrated male pig, with no apparent significant differences from other pigs of his age and category. The animal was raised in an intensive environment; the pig spent the first 1.5 months in a semi-open environment and was then moved to an enclosed environment. The daily feed consisted of feed from the farm feed mill, mainly corn, barley, bran, wheat, and soybean, with vitamin supplementation. No clinical signs were observed and the animal was found dead at 4 months (120 days) of age. No relevant clinical signs or changes in feeding behavior or activity of the pig had been reported prior to death. No exposures to toxic substances or relevant environmental factors were reported in the geographical area of the farm. The animal was submitted to the Istituto Zooprofilattico Sperimentale dell’Umbria e delle Marche (IZSUM) “Togo Rosati” for post-mortem examination.

### Anatomopathological and immunohistochemical examination

**Fig. 1:**
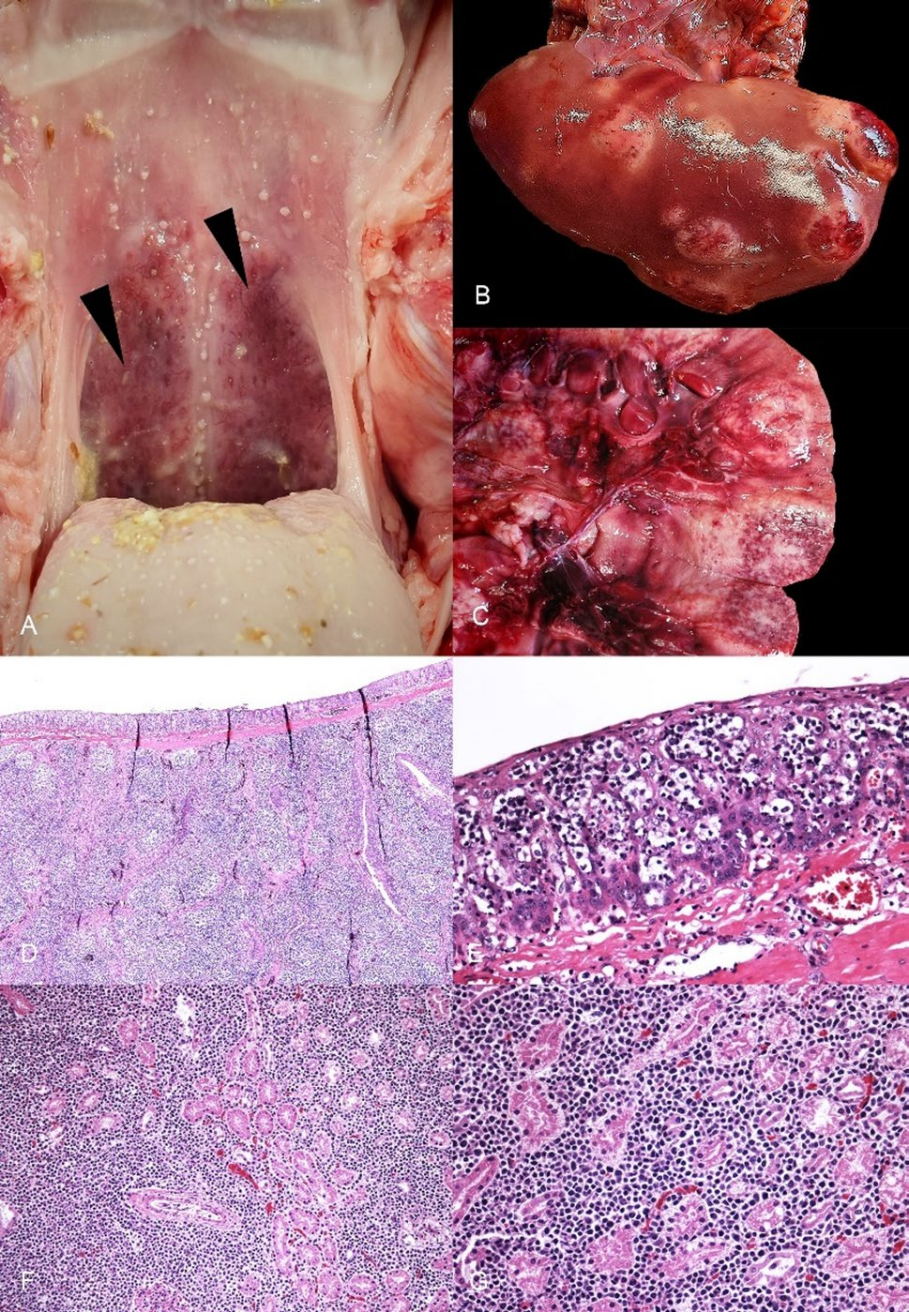
Macroscopic and histologic features of tonsillar lymphoma and hepatic and renal metastases in a domestic pig. (**A**) Soft palate tonsils showing irregular surface and red color; (**B**) Liver characterized by multiple white to gray nodules; (**C**) Kidney showing the cut surface of white metastatic nodules; (**D**) Soft palate tonsils largely expanded by a multinodular proliferation of lymphoid tissue (H&E; x40); (**E**) Soft palate tonsils with intraepithelial aggregates of neoplastic cells (Pautrier’s microabscesses) almost diffusely obscuring the lining mucosal epithelium (H&E; x400); (**F**) Renal parenchyma with a diffuse proliferation of sheets of round cells (H&E; x200); (**G**) Renal parenchyma with a monomorphic proliferation of medium to large neoplastic round cells (H&E; x400)

A complete autopsy was performed at the IZSUM. On external examination, the animal showed no obvious lesions. Good body condition and animal preservation were noticed. On examination of the oral cavity and pharynx, marked enlargement of the tonsils of the soft palate was observed, with firm consistency and a red color (Fig. 1A). On opening the abdominal cavity, multiple, white-to-gray, generally well-demarcated nodules reaching 6 cm in diameter were identified in liver and kidney (Fig. 1B-C). No abnormalities were noticed in other organs, including lymph nodes, thymus and spleen. At this stage a neoplastic process was suspected macroscopically; bacteriological and virological tests were not performed. Samples of the soft palate tonsils, liver and kidney were fixed in in 10% neutral buffered formalin. Subsequently, based on standard protocols, formalin-fixed paraffin embedded samples were cut into 3–5 μm sections, and stained with Hematoxylin and Eosin stain (H&E). The histology sections examined revealed that the tonsillar lymphoid tissue of the soft palate was largely expanded by a multinodular proliferation of medium to large round cells (diameter larger than 1.5–2 red blood cells). These cells were characterized by round or oval nuclei, with finely scattered chromatin, prominent nucleoli, and scant, basophilic cytoplasm. Mitotic count was equal to 4 mitotic figures on 2.37mm2 (10 fields x40, FN22); widespread apoptosis was observed. Numerous aggregates of large neoplastic cells were detected in the tonsillar epithelium (Pautrier’s microabscesses). The same cell population was also present in the liver and kidney, where round neoplastic cells invaded and replaced the normal parenchyma, confirming the metastatic nature of the process.

**Fig. 2:**
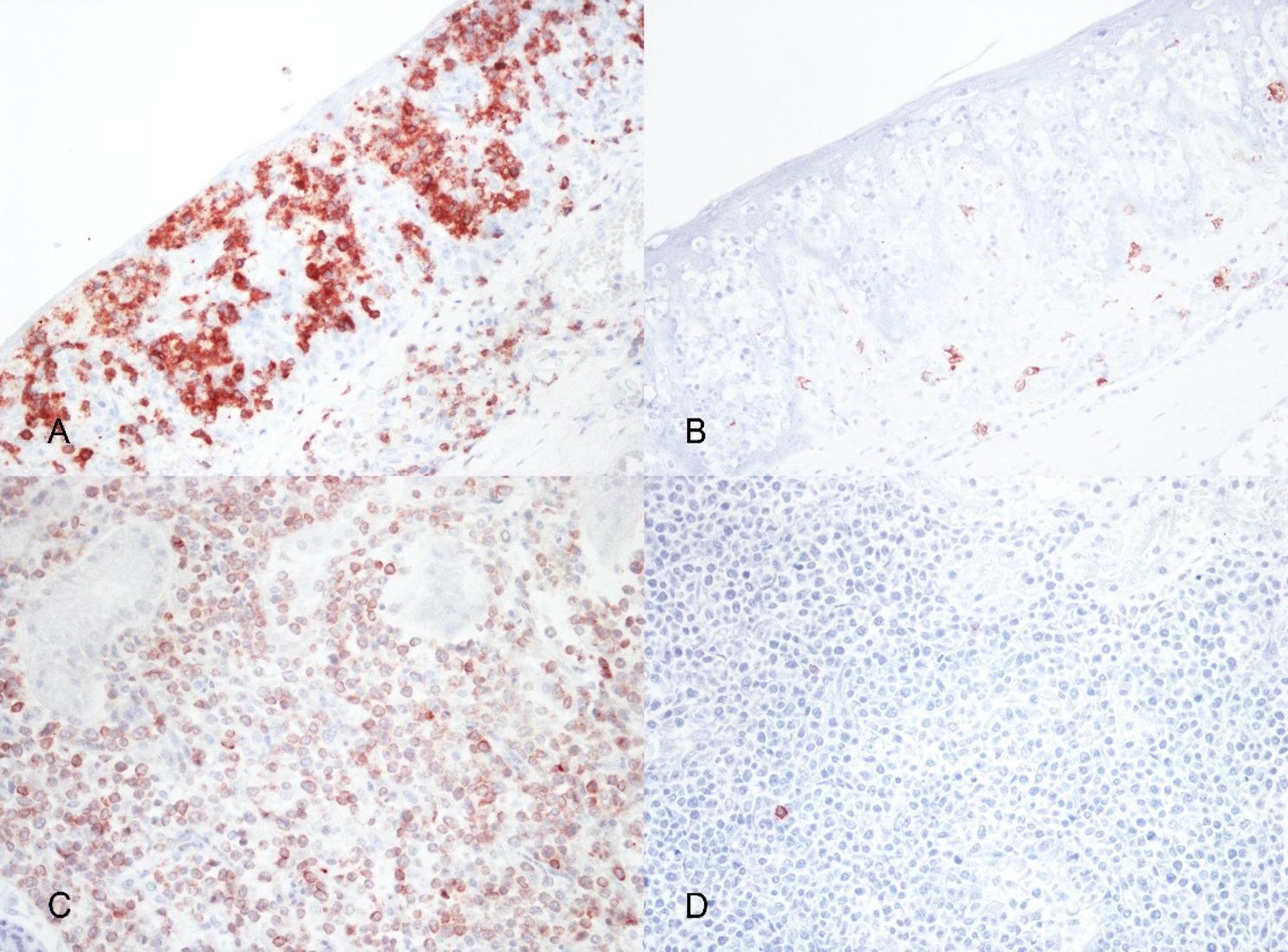
Immunohistochemical phenotyping of tonsillar lymphoma and renal metastases in a domestic pig. **A** Soft palate tonsils epithelium showing numerous CD3-positive cells in intraepithelial aggregates (CD3 IHC; x400); (**B**) Soft palate tonsils epithelium showing rare reactive CD20-positive cells (CD20 IHC; x400); (**C**) Kidney parenchyma showing numerous CD3-positive infiltrating neoplastic cells (CD3 IHC; x200); (**D**) Kidney parenchyma showing rare reactive CD20-positive cells (CD20 IHC; x200)

To better characterize the nature of the neoplastic cells, immunohistochemical tests with lymphoid cell-specific markers were performed. Immunolabeling was performed using anti-CD3 and anti-CD20 antibodies. The CD3 marker was detected using a rabbit polyclonal antibody (Dako), while the CD20 marker was identified using a rabbit polyclonal antibody (ThermoFisher Scientific). In both cases, antigen retrieval treatment with TRIS-EDTA buffer pH 9.0 was performed. Incubations were conducted at room temperature: anti-CD3 antibody was diluted 1:200 and incubated for 1-hour, anti-CD20 antibody was diluted 1:200 with an incubation time of 2 h. Results of the immunohistochemistry showed a widespread positivity in the neoplastic cells for CD3 (T-lymphocytic origin) (Fig. 2A) with rare CD20 positive reactive B-lymphocytes (Fig. 2B). The same CD3-positive neoplastic cells were detected in the Pautrier’s microabscesses and in the metastases in liver and kidney (Fig. 2C) with scattered reactive B-lymphocytes (Fig. 2D). On the basis of macroscopic, histologic and immunophenotypic features, a diagnosis of primary tonsillar T-cell lymphoma with liver and kidney metastasis was made.

## Discussion and conclusion

Lymphoma represents the most common neoplasm of the hematopoietic system, which is characterized by the uncontrolled proliferation of lymphoid cells at different levels of differentiation, and can affect different anatomical sites [1, 2, 5]. In pigs, lymphoma is documented sporadically in the veterinary literature but is reported from some studies as the most frequent form of hematopoietic tumor in this species. According to the systematic review conducted by Vasconcelos et al., in 2023, which analyzed the incidence of neoplasms in pigs and domestic ruminants, lymphomas account for 19.2% of all neoplasms observed in pigs, representing a significant proportion among neoplastic diseases and second most common category of neoplasia in this species [1]. The anatomical distribution of these neoplasms is predominantly associated with the hematopoietic system, but cases with involvement of other systems were also reported [1, 5].

Compared with other livestock species, such as cattle, lymphoma in pigs has a significantly lower incidence [21]. Also in small domestic animals, particularly in dogs and cats, lymphoma is the most common hematopoietic neoplasm, frequently diagnosed and subject of extensive and deep characterization [22]. In pigs, despite its low incidence, lymphoma can occur at any age [1, 2, 23]. This occurrence raises the hypothesis of a potential relation to genetic mutations. Although hereditary occurrence is reported in the literature in the development of porcine lymphomas, it was not investigated in the present case [23]. The failure to perform genetic investigations is a potential limitation of the study, as it prevents a thorough evaluation of possible underlying pathogenetic mechanisms. However, no other similar cases have been reported in subjects of the same litter, making hereditary occurrence less likely in the current case. Additionally, regarding the lymphomagenesis, as a component of the mucosa-associated lymphoid tissue, the tonsil is continuously exposed to environmental and microbial antigens, potentially resulting in sustained antigen exposure and immune stimulation. Persistent antigenic stimulation and antigen-driven inflammation has been implicated in the development of human lymphomas and could have played a potential role in the present case [24].

Clinically, no specific signs have been reported in cases of porcine lymphoma and cases with sudden death and no premonitory signs are reported [2]. However, in intensive production systems, finishing pigs are generally sent to slaughter around six months of age without being subjected to extensive health monitoring unless there are obvious clinical signs or evident impairment of animal development. In addition, sudden mortality is often attributed to infectious, metabolic, or management-related diseases, and is not always followed by full-autopsy. This approach severely limits the possibility of identifying lymphoid tumors that, as in the case described, may progress asymptomatically to the terminal stage. Considering this, it is likely that the true incidence of lymphoma in pigs is possibly underestimated, and only a limited proportion of cases are recognized and documented in literature.

In the case described in this report, the pig did not exhibit any relevant clinical signs in the days or weeks prior to death. The animal was regularly fed, showed behavior in line with growth standards for individuals of the same age and production category, with no obvious developmental delays. The absence of clinical signs and sudden death make the clinical picture of particular interest, as the presence of tonsillar lymphoma with liver and kidney metastases was detected only at necropsy. This suggests a key role of post-mortem investigation in all the cases of sudden death in pigs.

Anatomopathologically, the lymphoma had an unusual primary location, the tonsils, a site not previously described as primary origin of lymphoid neoplasm in pigs. The tonsils appeared macroscopically enlarged and red, with irregular surface. Histologic examination and immunophenotyping with immunohistochemistry confirmed the presence of a neoplastic proliferation of T-lymphocytes. The presence of neoplastic nodules in the liver and kidney, in absence of involvement of other lymphoid organs, supported a systemic dissemination of the neoplastic process, rather than a multicentric origin, suggesting also that the disease was already at an advanced stage at the time of death. Similar cases of primary T-cell tonsillar lymphoma in pigs are not documented in the literature, making this case of particular interest.

The occurrence in a young, apparently healthy pig presenting with sudden death underscores the need to include lymphomas in the differential diagnosis in cases of sudden death. Additionally, from a practical point of view, the case emphasizes the need of careful examination of the tonsils during post-mortem examination and inspection at slaughter, especially in presence of lesions compatible with liver and renal metastases, to increase the chance of detecting the primary site of development. Tonsillar enlargement in pigs is most often interpreted as inflammatory or hyperplastic and commonly associated with infectious agents, including those of zoonotic relevance (e.g., *Streptococcus suis*) [25]. As demonstrated by this case, however, primary tonsillar lymphoma represents an unusual but potential diagnosis, as neoplastic lesions may closely mimic chronic proliferative non-neoplastic processes and lead to underdiagnosis of the primary site of tumor development. It also suggests the usefulness of implementing systematic reporting systems for cancerous lesions in pigs to improve health surveillance and deepen the epidemiology of neoplasms in the swine species. Increased awareness among farm veterinarians, inspectors, and diagnosticians can help refine early diagnosis and health management on the farm.

## Data Availability

All data generated during this study are included in this published article.
